# Histoplasmosis in an elderly polish tourist – a case report

**DOI:** 10.1186/s12890-019-0914-7

**Published:** 2019-08-14

**Authors:** Krzysztof Specjalski, Karolina Kita, Krzysztof Kuziemski, Beata Tokarska, Lucyna Górska, Jolanta Szade, Alicja Siemińska, Marta Chełmińska, Ewa Jassem

**Affiliations:** 1 Department of Allergology, Medical University of Gdańsk, ul. Dębinki 7, 80-952 Gdańsk, Poland; 2 Department of Radiology, Medical University of Gdańsk, Gdańsk, Poland; 3 Department of Pathology, Medical University of Gdańsk, Gdańsk, Poland

**Keywords:** Histoplasmosis, Fungal infections, Pulmonary infections, Histoplasma, Case report

## Abstract

**Background:**

Histoplasmosis is a mycosis caused by soil-based fungus *Histoplasma capsulatum* endemic in the USA, Latin America, Africa and South-East Asia. The disease is usually self-resolving, but exposure to a large inoculum or accompanying immune deficiencies may result in severe illness. Symptoms are unspecific with fever, cough and malaise as the most common. Thus, this is a case of disease which is difficult to diagnose and very rare in Europe. As a result, it is usually not suspected in elderly patients with cough and dyspnea.

**Case presentation:**

This is a case of a 78-year-old patient, admitted to our department due to respiratory failure, cough, shortness of breath, fever and weight loss with no response to antibiotics administered before the admission. Chest CT revealed numerous reticular and nodular infiltrations with distribution in all lobes. The cytopathology of BAL showed small parts of mycelium and numerous oval spores. Considering clinical presentation and history of travel to Mexico before onset of disease, pulmonary histoplasmosis was diagnosed. After introduction of antifungal treatment rapid improvement was achieved in terms of both clinical picture and respiratory function.

**Conclusions:**

Since the risk of *Histoplasma* exposure in Europe is minimal, patients, who present with dyspnea, fever and malaise are not primarily considered for diagnosis of histoplasmosis. However, taking into account increasing popularity of travelling, also by elderly or patients with impaired immunity, histoplasmosis should be included into differential diagnosis.

## Background

Histoplasmosis is a mycosis caused by soil-based fungus *Histoplasma capsulatum,* endemic in the USA (eg. Mississippi Valley and Ohio Valley), Latin America, Africa, India and Malaysia. Infection results from inhalation of the fungal spores present in organic matter enriched with bat and bird droppings. In virtually all cases the infection is asymptomatic or mildly symptomatic. The disease is usually self-resolving, but exposure to a large inoculum or accompanying immune deficiencies may result in severe illness. The main defense against *Histoplasma capsulatum* is cellular response. Hence, in elderly patients or individuals with defective T- cell immunity, such as AIDS, infection may prolong and have a severe course [1]. In the endemic regions histoplasmosis is usually diagnosed in farmers, construction or demolition workers, tourists and spelunkers. In the USA, the majority of 2850 cases recorded between 1938 and 2013 were related to demolition of old buildings or working in the chicken coops [2].

## Case presentation

A 78-year-old patient, family doctor, was admitted to our department due to cough, shortness of breath, fever, loss of appetite associated with 10 kg weight loss, and night sweating. These symptoms were deteriorating within the three proceeding months. Past history included diabetes mellitus type 2 and benign prostatic hypertrophy.

**Fig. 1:**
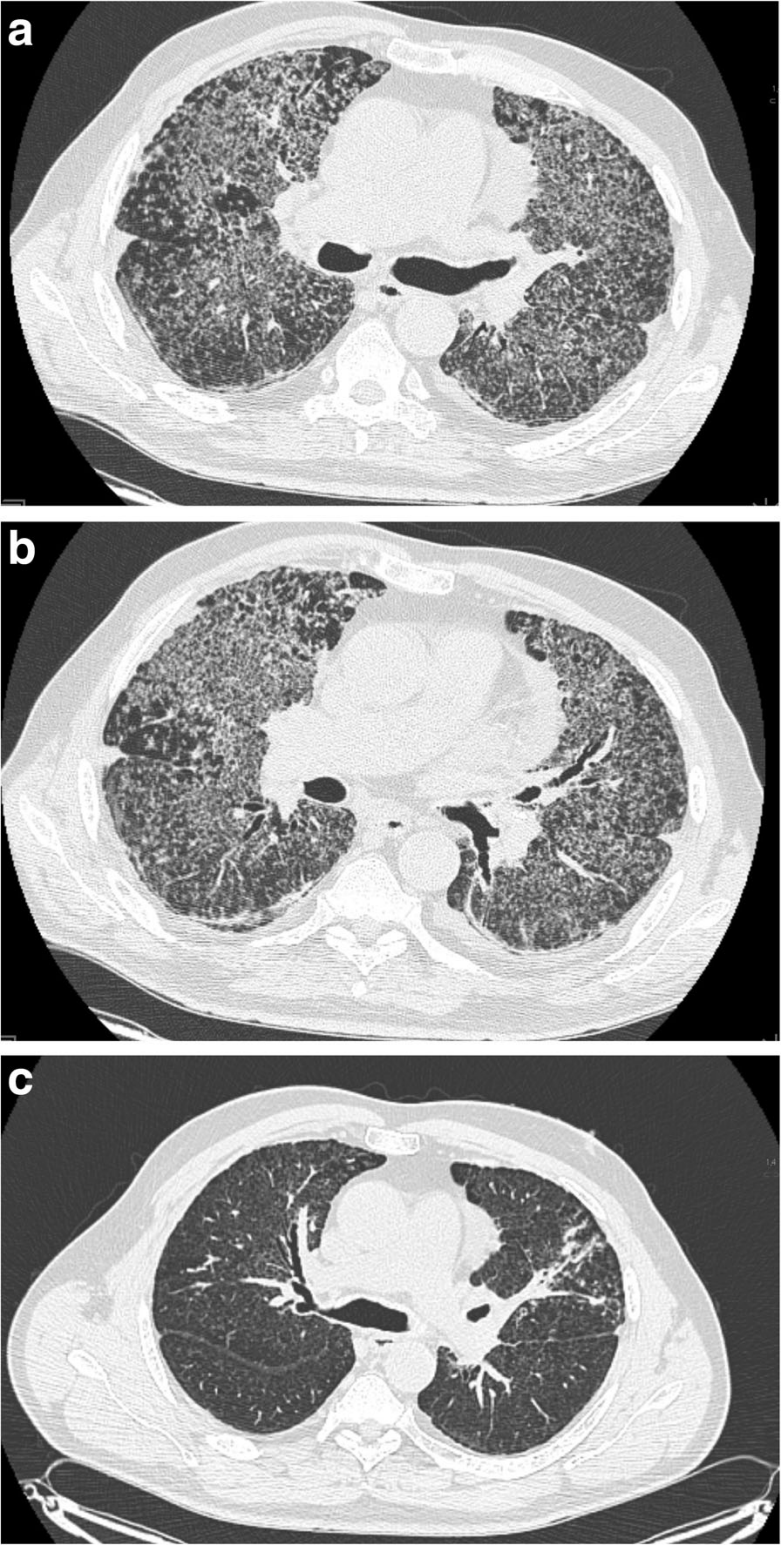
CT scans during first hospitalization (**a**), on admission to our department (**b**) and at check-up visit 1 year later (**c**)

Two weeks before the onset of the disease the patient visited Mexico and was exploring caves. However, as far as we know, he was the only participant who developed respiratory symptoms after the trip. Considering the risk of a tropical disease, as soon as the first symptoms appeared, he was admitted to the Centre of Tropical Diseases. At admission blood markers of inflammation (CRP, PCT, leukocytosis) were elevated, however blood and urine cultures were negative (Table 1). The tests for malaria, dengue, parasitic infections and HIV were negative. Chest CT revealed bilateral small nodular opacities, interlobular septal thickening, and a single irregular opacity (20 X 17 mm) with cavitation and calcified hilar lymph node in the left lung (Fig. 1a). Patient received amoxicillin with clavulate and clarithromycin, followed by ceftriaxone and fluconazole. Since no response was obtained, he was transferred to the lung diseases centre for the further diagnostic tests. During this hospitalization bronchoscopy was performed. It showed bronchial purulent inflammation with easily bleeding mucosa. BAL was not performed, but the aspirated bronchial secretion was used for culture. It was positive for *Citrobacter freundi* and, as a consequence, sulfamethoxazole with trimethoprim was administered. Since no clinical improvement was achieved, the patient was subjected to open lung biopsy. Pathologic examination showed lymphocytic infiltrations, peribronchial granulomas with giant cells and mild fibrosis, leading to the diagnosis of allergic extrinsic alveolitis. The patient was administered oral steroids (40 mg prednisone daily) with no clinical response.

**Table 1:** Laboratory findings during first hospitalization (1), on admission to the Department of Allergology and Pneumonology (2), 3rd day after admission (3), after introduction of antifungal therapy (4), on discharge (5), on follow-up visit after first month of treatment (6)

	(1)	(2)	(3)	(4)	(5)	(6)
CRP (mg/l)	**29**	**25**	**41.1**	**27.9**	0.73	0.61
Creatinine (mg/dl)	0.82	0.7	0.67	0.81	0.96	1.07
Bilirubin (mg/dl)	0.6	0.78	0.54	0.36	0.47	0.48
ALT (U/l)	25	52	39	29	53	38
AST (U/l)	13	29	14	11	24	17
GGTP (U/l)	**80**	**103**	**86**	51	**101**	**85**
D-dimer (μg/l)	**902**	**551**				
LDH (U/l)	**317**	**269**				
ALP (U/l)	55	77	67	37	49	52
Na (mmol/l)	136	133	137	140	138	136
K (mmol/l)	4.3	4.9	3.7	5.1	4.0	4.2
Hb (g/dl)	14.5	15.0	14.8	**12.8**	**11.4**	13.8
WBC (G/l)	7.37	7.28	7.98	**10.61**	8.82	6.91
GRAN (G/l)	5.57	5.68	5.51	**8.68**	4.85	4.29
LYMP(G/l)	**0.94**	**0.75**	1.48	1.13	**0.91**	1.47
PLT (G/l)	207	224	271	**134**	160	166
pH	7.43	7.44	7.43	7.46	7.38	7.46
pCO_2_ (mmHg)	34.9	29.1	32.4	34.0	32.8	31.6
pO_2_ (mmHg)	79.9	73.6	44.5	61.6	66.5	68.7
Blood culture	Negative	negative				
Sputum culture		normal flora				
Aspergillus galactomannan		negative				
Candida mannan		negative				
*Pneumocystic jiroveci* – direct IF		negative				
HIV		negative				
CMV antigen pp65		negative				
ANA-Hep2		negative				
**(2)**	BAL cytology: macrophages – 52%; neutrophils – 8%; lymphocytes – 35%; monocytes – 3%; eosinophils – 2%

Nearly 3 months after the first symptoms appeared the patient was admitted to the Department of Allergology and Pneumonology, Medical University of Gdansk. On admission dominating symptoms included breathlessness, poor tolerance of physical activity, malaise and sweating, which were accompanied by body temperature of 38 °C. At auscultation bilateral basal crackles were found. Laboratory tests revealed moderately elevated CRP with no leukocytosis (Table 1). Patient’s condition was deteriorating day by day, with respiratory failure requiring oxygen supplementation from the third day of hospitalization. As the patient’s condition required making a diagnosis fast, we decided to perform wide range of lab tests, repeat chest CT and bronchoscopy with BAL. On the chest CT numerous reticular and nodular infiltrations were found, with central and peripheral distribution in all lobes widely spread in both lungs – very similar to the first CT scans (Fig. 1b). In BAL cytology macrophages and lymphocytes dominated (Table 1). BAL cultures for both bacteria and fungi were negative. However, microscopy revealed small part of mycelium and numerous oval spores resembling *Histoplasma capsulatum* or *Pneumocystis jiroveci*. Genetic identification of pathogen was not available in our hospital. The blood sample was sent for serology.

Considering patient’s serious condition and high probability of histoplasmosis (clinical presentation, travel to endemic area, suspicion of fungal infection in microscopy and no response to antibiotics), the patient received liposomal amphotericin (300 mg i.v. daily i.e. 5 mg/kg for 14 days) followed by itraconazole (600 mg daily for 3 days, then 400 mg daily for 3 months). Dyspnoea and fever substantially resolved during the first week of treatment. At that time we received positive result of serology (positive IgM, borderline IgG) finally confirming suspicion of histoplasmosis and justifying the treatment. After 3 weeks the patient was discharged from the hospital. Two months after the discharge in follow up serology IgM became negative and IgG – positive. Two year follow-up showed no recurrence of signs and symptoms.

The participant has given written consent to take part in the case report.

## Discussion and conclusions

The circumstances of the disease onset in our patient were quite typical. Most probably, he was infected in Mexico while exploring caves. Since the risk of *Histoplasma* exposure in Europe is minimal, patients, who present with dyspnea, fever and malaise are not primarily considered for diagnosis of histoplasmosis. Remarkably however, in the recent series from Israel all cases of histoplasmosis were associated with travelling to endemic areas and visiting caves or other places inhabited by bats [3]. Thus, a thorough traveling history should be recommended in suspected individuals.

Despite unspecificity of symptoms, fever, cough and malaise occur in 45, 42 and 34% of patients with histoplasmosis, respectively. These symptoms are often accompanied by chest pain, myalgia, sweating, chills and weight loss [1].

Histoplasmosis can be confirmed by identifying the fungus in lung biopsy or BAL fluid samples, detection of antigen by serology and genetic tests or antibodies against respective antigens. According to the criteria of the European Organization for Research and Treatment of Cancer / Invasive Fungal Infections Cooperative Group and the National Institute of Allergy and Infectious Diseases Mycoses Study Group (EORTC/MSG) a proven diagnosis is contingent on confirmation by either histopathology or culture, while a probable diagnosis is based on the typical clinical picture, predisposing condition and mycologic evidence (e.g. antigenuria) [4]. Sensitivity of each method depends on the clinical manifestation (pulmonary vs. disseminated), the state of patient’s immunity and the burden of the disease. In acute pulmonary histoplasmosis sensitivity of culture and pathology is low (< 20 and < 42%, respectively) with higher number of positive results of serology (approx. 65%) and antigen detection (approx. 83%) [5]. Some authors recommend application of serologic tests and pathology together to achieve the highest accuracy [6]. Skin tests, once commonly used, have been abandoned due to high background positivity in endemic areas [7].

The final diagnosis of histoplasmosis is often delayed due to lack of specific clinical features and necessity of wide differential diagnosis. In our patient time elapsed from first symptoms to commencement of appropriate therapy was more than 3 months. That was probably related to the fact that frequent travelling by elderly patients to exotic places is relatively new phenomenon in Poland. Thus, epidemiology of tropical diseases and endemic pathogens are not widely known and they are not considered in differential diagnosis. As a result of delayed diagnosis proportion of patients receive unnecessary treatment for common bacterial infections or tuberculosis [8]. Our patient received several antibiotics what may have led to superinfection and another antibiotic therapy. Making the diagnosis was quite difficult as experience in diagnosing and treating histoplasmosis was limited in Poland and some diagnostic tests (antigen detection, molecular methods) were not available. Moreover, the culture was negative what may be explained by the low sensitivity of this method described before [5]. What helped us to confirm the suspicion of histoplasmosis was serology. Its positivity results from an exposure to the fungus at some point in the past, so it should be interpreted cautiously. However, considering the clinical manifestation, recent travel to the endemic area and the lack of exposure in the homeland, positive IgM was interpreted as a reliable confirmation of histoplasmosis in our patient.

In moderately severe or severe forms of pulmonary histoplasmosis, Infectious Diseases Society of America recommends the use of liposomal amphotericin B (3.0–5.0 mg/kg qd intravenously for 1–2 weeks) followed by itraconazole (200 mg tid for 3 days and then 200 mg bid for total of 12 weeks). In patients with low risk of nephrotoxicity alternative therapy includes deoxycholate formulation of amphotericin. Additionally, methylprednisolone during the first 1–2 weeks of antifungal therapy is recommended in patients who develop respiratory complications, including hypoxemia or significant respiratory distress [9]. Prognosis is good, unless the treatment is inadequate.

To conclude, histoplasmosis should be included into differential diagnosis in patients presenting with dyspnea and cough after travelling to endemic areas.

## Data Availability

The presented data are available from the corresponding author on reasonable request.

## Abbreviations

ALP: Alkaline phosphatase
ALT: Alanine aminotransferase
AST: Aspartate aminotransferase
BAL: Bronchoalveolar lavage
CRP: C-reactive protein
CT: Computed tomography
GGTP: Gamma-glutamyl transferase
LDH: Lactate dehydrogenase
PCT: Procalcitonin
